# Subcellular Localization of Acyl-CoA: Lysophosphatidylethanolamine Acyltransferases (LPEATs) and the Effects of Knocking-Out and Overexpression of Their Genes on Autophagy Markers Level and Life Span of *A. thaliana*

**DOI:** 10.3390/ijms22063006

**Published:** 2021-03-16

**Authors:** Katarzyna Jasieniecka-Gazarkiewicz, Kamil Demski, Satinder K. Gidda, Sylwia Klińska, Janusz Niedojadło, Ida Lager, Anders S. Carlsson, Elena A. Minina, Robert T. Mullen, Peter V. Bozhkov, Sten Stymne, Antoni Banaś

**Affiliations:** 1 Intercollegiate Faculty of Biotechnology, University of Gdansk and Medical University of Gdansk, 80-307 Gdansk, Poland; kamil.demski@biotech.ug.edu.pl (K.D.); sylwia.klinska@phdstud.ug.edu.pl (S.K.); antoni.banas@biotech.ug.edu.pl (A.B.); 2 Department of Molecular and Cellular Biology, University of Guelph, Guelph, ON N1G 2W1, Canada; sgidda@uoguelph.ca (S.K.G.); rtmullen@uoguelph.ca (R.T.M.); 3 Department of Cell Biology, Department of Cellular and Molecular Biology, Nicolaus Copernicus University, 87-100 Torun, Poland; janiaszn@umk.pl; 4 Department of Plant Breeding, Swedish University of Agricultural Sciences, 230-53 Alnarp, Sweden; ida.lager@slu.se (I.L.); anders.carlsson@slu.se (A.S.C.); sten.stymne@slu.se (S.S.); 5 Department of Molecular Sciences, Uppsala BioCenter, Swedish University of Agricultural Sciences and Linnean Center for Plant Biology, 750-07 Uppsala, Sweden; alena.minina@slu.se (E.A.M.); peter.bozhkov@slu.se (P.V.B.)

**Keywords:** autophagy, NBR1, ATG8, acyl-CoA:lysophosphatidylethanolamine acyltransferases, *Arabidopsis thaliana*, senescence

## Abstract

*Arabidopsis thaliana* possesses two acyl-CoA:lysophosphatidylethanolamine acyltransferases, LPEAT1 and LPEAT2, which are encoded by *At1g80950* and *At2g45670* genes, respectively. Both single *lpeat2* mutant and double *lpeat1 lpeat2* mutant plants exhibit a variety of conspicuous phenotypes, including dwarfed growth. Confocal microscopic analysis of tobacco suspension-cultured cells transiently transformed with green fluorescent protein-tagged versions of LPEAT1 or LPEAT2 revealed that LPEAT1 is localized to the endoplasmic reticulum (ER), whereas LPEAT2 is localized to both Golgi and late endosomes. Considering that the primary product of the reaction catalyzed by LPEATs is phosphatidylethanolamine, which is known to be covalently conjugated with autophagy-related protein ATG8 during a key step of the formation of autophagosomes, we investigated the requirements for LPEATs to engage in autophagic activity in Arabidopsis. Knocking out of either or both *LPEAT* genes led to enhanced accumulation of the autophagic adaptor protein NBR1 and decreased levels of both *ATG8a* mRNA and total ATG8 protein. Moreover, we detected significantly fewer membrane objects in the vacuoles of *lpeat1 lpeat2* double mutant mesophyll cells than in vacuoles of control plants. However, contrary to what has been reported on autophagy deficient plants, the *lpeat* mutants displayed a prolonged life span compared to wild type, including delayed senescence.

## 1. Introduction

Acyl-CoA:lysophosphatidylethanolamine acyltransferases (LPEAT) belong to a broad family of enzymes (acyl-CoA:lysophospholipid acyltransferases; LPLAT) which utilise acyl-CoAs as fatty acid donors and lysophospholipids as fatty acid acceptors producing the appropriate phospholipids [1]. In *Arabidopsis thaliana,* there are two genes encoding LPEATs: *LPEAT1* (At1g80950) and *LPEAT2* (At2g45670) [2]. Until recently, LPEATs were considered (similarly to other LPLATs) to be primarily enzyme constituents of the Lands’ Cycle and involved together with phospholipase A2 (PLA2) in phosphatidylethanolamine (PE) remodeling [3]. However, recent studies have shown that LPEAT can remodel PE and, to some extent, other phospholipids without the involvement of PLA2 via the reverse reaction [4]. Additionally, it has been shown recently that LPEAT is potentially involved in the regulation of *A. thaliana* plant growth [5]. For instance, *LPEAT2* T-DNA knock-out mutant plants displayed a particularly strong phenotype, including having an overall dwarfed appearance, with smaller leaves, fewer seeds, and suppressed root growth as compared to wild-type plants. This phenotype was also accompanied by significantly lower levels of PE, especially species with very-long-chain fatty acids (PE40C and PE42C), as well elevated levels of lysophosphatidylethanolamine (LPE) and lysophosphatidylcholine (LPC) compared to the wild-type plants [5]. By comparison, *lpeat1* knock-out mutant plants displayed a similar, but less pronounced phenotype, whereas the corresponding *lpeat1 lpeat2* double mutant plant was more similar to the *lpeat2* mutant, suggesting that LPEAT2 is the more predominant isoform. In contrast to the observations reported for the loss-of-function mutants, plants that overexpressed *LPEAT1* or *LPEAT2* were relatively larger and produced more seeds than wild type plants [5]. Notably, all of the abovementioned effects were attributed specifically to LPEATs, since disruption or overexpression of other members of the LPLAT family in Arabidopsis, namely the acyl-CoA:lysophosphatidylcholine acyltransferases (LPCAT), did not result in any obvious phenotypic effects in plant growth and development [6].

The main product of LPEAT activity, PE, is the second most abundant phospholipid (after phosphatidylcholine) in eukaryotic cells [7]. The Kennedy Pathway is the primary pathway of PE synthesis, whereby diacylglycerol (DAG) is converted into PE by CDP-ethanolamine:1,2-diacylglycerol ethanolaminephosphotransferase, which utilizes the active form of ethanolamine (CDP-ethanolamine) and DAG [8]. PE is a membrane lipid and can be converted in different ways to LPE, including by reactions catalyzed by PDATs (phospholipid: diacylglycerol acyltransferases) or phospholipases. The resulting LPE is subsequently converted again to PE in reaction catalyzed by LPEATs (acyl-CoA: lysophosphatidylethanolamine acyltransferases) or can be further degraded [9]. Consequently, the disruption of the *LPEAT* genes in *A. thaliana* (see above) is considered to lead to a reduction in PE and a concomitant increase in LPE [5]. Thus, it seems that other LPLATs (acyl-CoA: lysophospholipid acyltransferases) cannot compensate for the loss of LPEAT activity in *lpeat* mutant plants and LPE is not fully reacylated leading to reduced PE levels.

Recently, it was reported that autophagosome formation during autophagy, a major catabolic process in eukaryotes, depends on PE concentration in *Saccharomyces cerevisiae* and *Drosophila melanogaster* [10]. Whether the abundance of PE regulates autophagic activity in plants, however, is unknown. Considering that the phenotypes of *lpeat* mutant plants [5] resemble, at least in part, those reported for autophagy-deficient mutants [11,12] and that the former also correlates with the decrease in PE content [5], we hypothesized that the mechanism of LPEAT-mediated regulation of plant growth might be due to its effect on autophagy. Here, we tested this hypothesis and also provide information regarding the subcellular localization of LPEAT1 and LPEAT2, as well as report on influence of *LPEAT* gene disruption or overexpression on the development of senescence in *A. thaliana*.

## 2. Results

### 2.1. Subcellular Localisation of Arabidopsis LPEAT1 and LPEAT2

**Figure 1:**
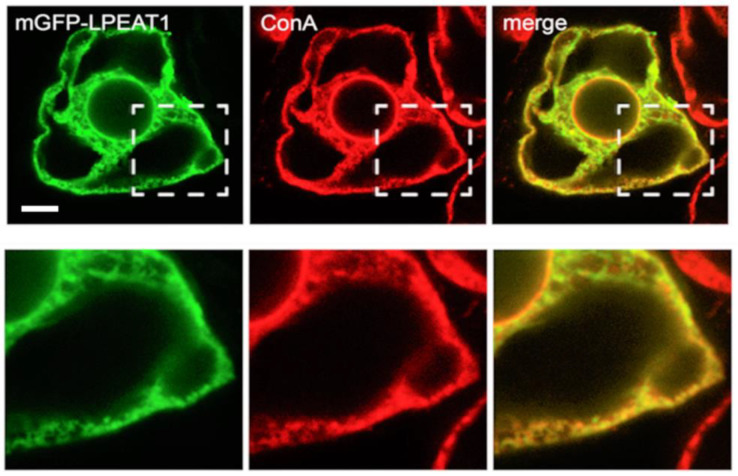
Subcellular localization of Arabidopsis LPEAT1 in BY-2 cells transiently-transformed via biolistic bombardment with mGFP-LPEAT1, formaldehyde-fixed and processed for confocal laser-scanning microscopy (CLSM), including staining with fluorescent dye-conjugated ConA, serving as an ER marker. Shown are representative images, as well as the corresponding merged image; hatch boxes represent the portion of the cell shown at higher magnification in the panels below. The yellow color in the merged image indicates co-localization between mGFP-LPEAT1 (green) and ConA (red) at the ER. Scale bar, 10 μm.

To investigate the subcellular localization of LPEAT1 and LPEAT2, we used transiently-transformed tobacco Bright Yellow-2 (BY-2) suspension-cultured cells as a well-established model system for studying plant protein localization in vivo [13,14,15]. As shown in Figure 1, monomerized green fluorescent protein (mGFP)-tagged LPEAT1 (mGFP-LPEAT1) yielded a distinct reticular localization pattern that matched that of the ER stain Concanavalin (ConA) [16]. These results indicate that LPEAT1 is localized to the ER, consistent with previous, large-scale plant organelle proteomics studies [17,18] and bioinformatic analyses of LPEAT1 at the Subcellular Localization Database for Arabidopsis Proteins (SUBA) [19]. 

**Figure 2:**
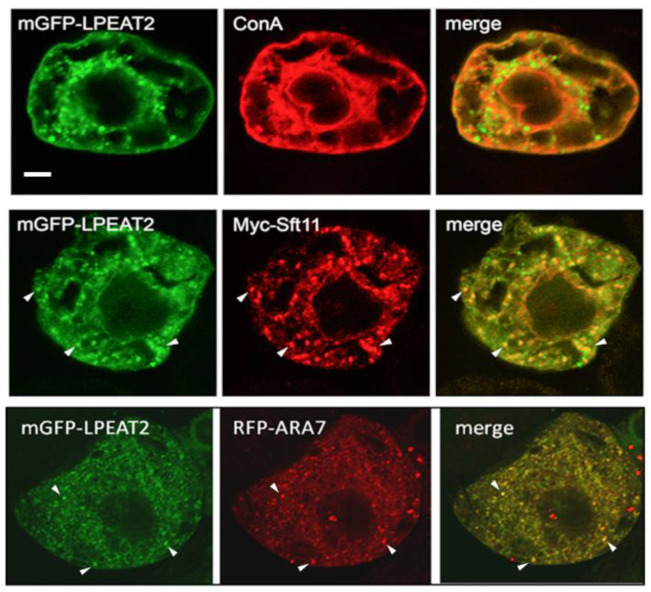
Subcellular localization of Arabidopsis LPEAT2 in BY-2 cells transiently-(co)transformed via biolistic bombardment with (as indicated by labels) mGFP-LPEAT2 (green) and the Golgi marker Myc-Sft11 (red) or the late endosomal marker RFP-ARA7 (red), or stained with fluorescent dye-conjugated ConA (red), serving as an ER stain. Shown are representative images, as well as the corresponding merged images for each set of (co)transformed cells. The yellow color in the merged images indicates co-localization of two proteins (also shown by arrowheads). Scale bar, 10 μm.

In contrast to LPEAT1, there is limited published data available on the subcellular localization of LPEAT2, which ranges from the protein being annotated (at SUBA) as a constituent of several different organelles, including the ER, Golgi, mitochondria, and nucleus, as well as the plasma membrane. As shown in Figure 2, mGFP-LPEAT2 did not as readily localize to the ConA-stained ER as compared to mGFP-LPEAT1 (Figure 1). However, mGFP-LPEAT2 did colocalize, albeit partially, with both Myc-eptitope tagged Arabidopsis SNARE (soluble *N*-ethylmaleimide-sensitive factor attachment protein receptor) Sft11, serving as a Golgi marker protein [20], and red fluorescent protein (RFP)-tagged Arabidopsis Rab5 (Rabenosyn-5)-related GTPase ARA7 (RFP-ARA7), serving a late endosome and multivesicular body marker protein [21,22] (Figure 2). These results suggest that LPEAT2 is localized to both Golgi and late endosomes, which are functionally related compartments of the endomembrane and endocytic pathways [23].

**Figure 3:**
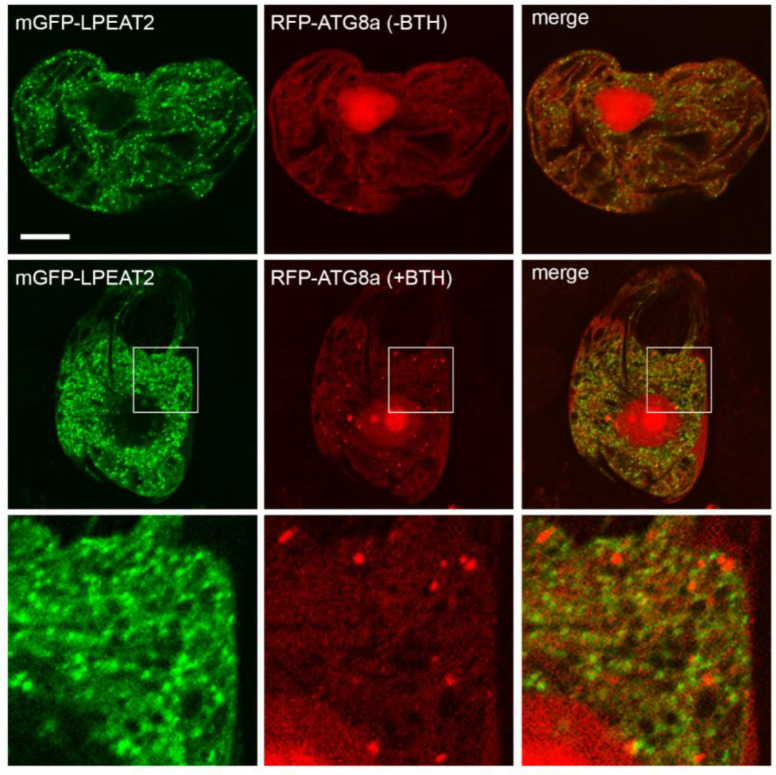
Arabidopsis LPEAT2 does not colocalize with the autophagosomal marker ATG8 in cells in the absence or presence of autophagy inducer BTH. BY-2 cells were transiently-cotransformed (as indicated by labels) with mGFP-LPEAT2 (green) and RFP-ATG8a (red) and incubated with or without 10 µM BTH. Cells were subsequently formaldehyde-fixed and processed for CLSM. Shown are representative images, as well as the corresponding merged images for each set of cotransformed cells. Boxes represent the portion of the cell co-expressing mGFP-LPEAT2 and RFP-ATG8a and incubated with BTH shown at higher magnification in the panels below. Note an increase in the number of RFP-ATG8-labelled puncta upon induction of autophagy with BTH indicating formation of autophagosomes. Note also a lack of colocalization between mGFP-LPEAT2 and RFP-ATG8a. Scale bar, 10 μm.

As mentioned in the Introduction, PE, the product of LPEAT activity, is involved in initiating autophagy in mammals and yeast through its conjugation to ATG8 (autophagy-related protein 8) [10]. Furthermore, both the ER and late endosomes have been implicated in autophagy pathways in plants, whereby the ER is thought to serve as the site(s) of autophagosome biogenesis and a platform for autophagy regulation [24], while late endosomes and autophagosomes share some proteins that mediate autophagic degradation [25]. These observations prompted us to examine the potential autophagosomal localization of LPEAT1 and LPEAT2 in plant cells upon induction of autophagy. As shown in Figure 3, mGFP-LPEAT2 did not display any obvious co-localization with the autophagosomal marker RFP-ATG8 in BY-2 cells treated with the salicylic acid agonist benzo-[1-3]-thiadiaole-7-carbonthioic acid S-methyl ester (BTH,) a potent autophagy inducer in plants ([26]; reviewed in Lin and Zhuang [27]). Similar results were obtained for LPEAT1 (data not shown) demonstrating that neither LPEAT isoform is targeted to autophagosomes during autophagy in plant cells.

### 2.2. NBR1 Transcript Levels and NBR1 Protein Abundance in Rosette Leaves of Wild-Type and LPEAT Transgenic Arabidopsis Plants

To investigate whether expression levels of LPEATs encoding genes and the products of their activity—LPEAT—might regulate autophagy, we evaluated the level of NBR1 (neighbor of BRCA1 gene) protein, a reporter of autophagic flux, in wild type plants and transgenic lines using Western blot analysis. In the preliminary experiments, we have shown that Arabidopsis NBR1 is a very labile protein and that the extracts can neither be stored before analyses nor the fresh leaves material can be used for extraction. Thus, the leaves of 4–5-week-old plants (0 DAF)-days after flowering were flash frozen in liquid nitrogen (100 mg; usually 1 to 2 leaves) and stored at −80 °C before being homogenized directly before analyses with 100 µl of extraction buffer. The proteins were directly denatured and separated on gels (for details see Materials and Methods). The above protocol resulted in good quality blots. 

**Figure 4:**
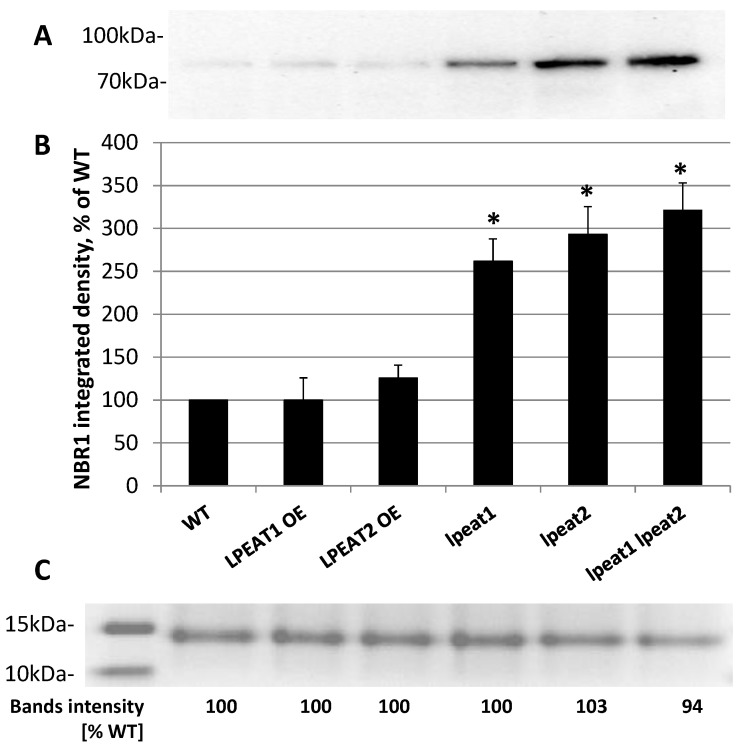
(**A**) NBR1-selected western blot analysis of the total protein extracts from leaves (0 DAF) of wild-type Arabidopsis (Col-0) and *LPEAT1* and *LPEAT2* overexpressors, *lpeat1* and *lpeat2* single mutants and *lpeat1 lpeat2* double mutant grown in soil under standard conditions. The blot was incubated with polyclonal antibodies against AtNBR1 protein. Equal amounts of protein (30 µg) were loaded and separated on NuPAGE 4–12% Bis-Tris-Gel. (**B**) NBR1 band average intensity (means ± SD) from 3 to 6 independent experiments as a percentage ofWT (wild type). Asterisk indicates significant difference compared with LPEAT1 OE (overexpressor) in “mean difference two sided test” at α = 0.05. (**C**) Fragment of selected gel with stained protein bands localized between 15 and 10 kDa and “Image Lab 6.01” analyses of the intensity of these bonds.

The amount of the NBR1 protein differed significantly between the wild-type Arabidopsis plants and tested *lpeat* mutants. On average, the levels of this protein in single (*lpeat1* and *lpeat2*) and double (*lpeat1 lpeat2*) mutants were 2.5–3 fold higher than in the wild-type plants (Figure 4). However, the differences between wild-type plants and *LPEAT* overexpressors were negligible (for the activity of LPEAT in tested mutants see Jasieniecka-Gazarkiewicz et al. [5]). 

**Figure 5:**
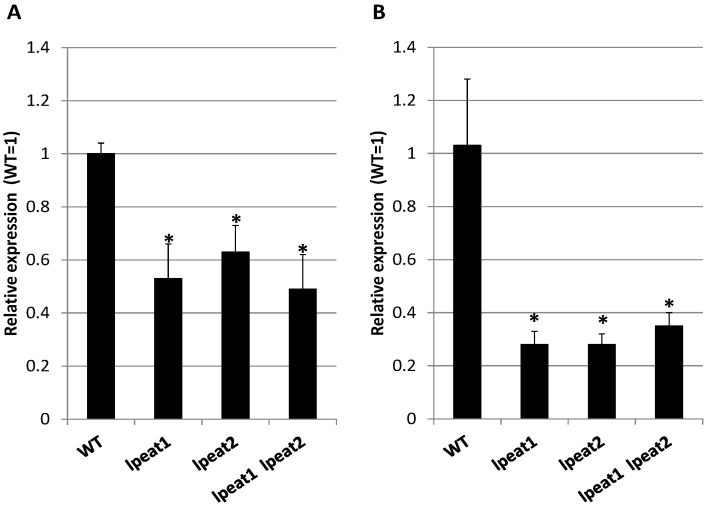
*NBR1* gene expression analysis between different *Arabidopsis thaliana* lines. *NBR1* transcript level in *lpeat1* mutant, *lpeat2* mutant and *lpeat1 lpeat2* double mutant is displayed as relative to this gene’s expression level in WT (wild-type) with the assigned value of 1. The *EF1ALPHA* (**A**) and *ACT2* (**B**) gene expression was treated as reference in qRT-PCR. Means ± SD are presented (n = 3). Asterisk (*) indicates significant difference compared with WT in “mean difference two sided test” at α = 0.05.

To verify that observed higher amount of NBR1 in *lpeat* mutants is not a result of higher expression of *NBR1* gene we compared the expression levels of *NBR1* in leaves of about four-week-old (0 DAF) wild type Arabidopsis plants and in leaves of *lpeat* mutants by qRT-PCR using *EF1ALPHA* (*At5g60390*) and *ACT2* (*At3g18780*) as reference genes. Normalizing the *NBR1* expression to the expression of both reference genes: *EF1ALPHA* (Figure 5A) and *ACT2* (Figure 5B) produced similar results. The expression levels of *NBR1* in *lpeat* mutant were lower than in the control plants. Thus, this results clearly showed that elevated levels of NBR1 in *lpeat* mutants are not a result of higher expression of *NBR1* but rather a result of lower degradation rate of this protein in the tested mutants. The conclusions concerning the degradation rate of NBR1 in control plants and in tested *lpeat* mutants should be, however, confirmed n further studies.

After separation on NuPAGE 4–12% Bis-Tris-Gel, the NBR1 protein was localized on the gels between 70 and 100 kDa proteins, as a one distinct band. The bands with lower intensity and less sharp structure were localized also below 40 kDa protein, and were treated as degradation products (when extracts were stored for some time in 4 °C only these bands were detected) (Figure S1). For the separation, equal amounts of protein (30 µg) were loaded on the gels. As an additional loading control simultaneously with the gel used for blotting analyses, we randomly separated the same denatured extracts on another gel and stained it directly after separation with “Commassie Brillant Blue” (example of full gel on Figure S5). The measurement of major bands intensity (on such stained gels) of separated extracts of tested Arabidopsis lines with “Image Lab 6.01” program revealed that the differences between the tested lines were negligible (an example on Figure 4).

### 2.3. ATG8a Transcript Levels and ATG8 Protein Abundance in Rosette Leaves of Wild-Type and LPEAT Transgenic Arabidopsis Plants

**Figure 6:**
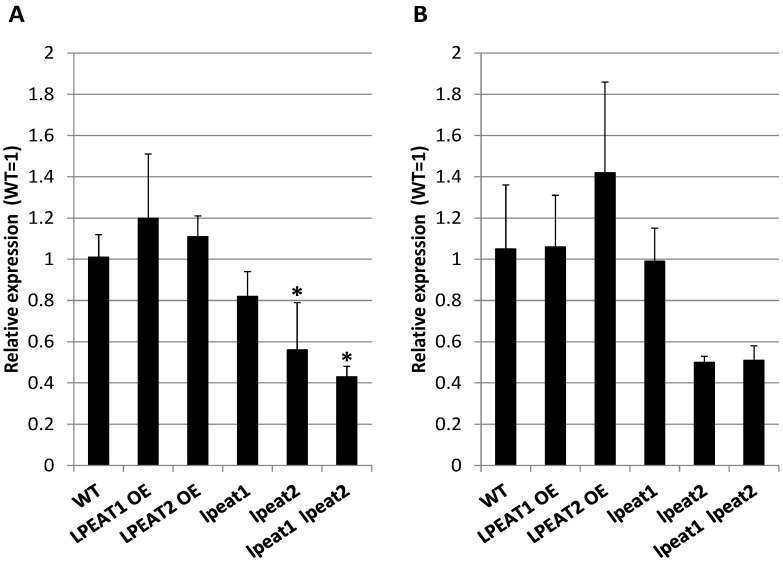
*ATG8a* gene expression analysis between different *Arabidopsis thaliana* lines. *ATG8a* transcript level in *LPEAT1* and *LPEAT2* overexpressors as well as in *lpeat1* mutant, *lpeat2* mutant and *lpeat1 lpeat2* double mutant is displayed as relative to this gene’s expression level in wild-type (WT) with the assigned value of 1. The *EF1ALPHA* (**A**) and *ACT2* (**B**) gene expression was treated as reference in qRT-PCR. Means ± SD are presented (n = 3). Asterisk indicates significant difference compared with WT in “mean difference two sided test” at α = 0.05.

ATG8 is the other protein, which could serve as an indicator of autophagy intensity [28,29,30]. There are nine isoforms (ATG8a–ATG8i) of ATG8 protein family in Arabidopsis [31]. In this study we compared the expression level of *ATG8a* in leaves of about four-week-old (0 DAF) wild type of Arabidopsis plants and in leaves of *lpeat* mutants and *LPEAT* overexpressors by qRT-PCR using *EF1ALPHA* (*At5g60390*) and *ACT2* (*At3g18780*) as reference genes. Normalizing the *ATG8a* expression to the expression of both reference genes: *EF1ALPHA* (Figure 6A) and *ACT2* (Figure 6B) produced similar results. In *LPEAT1* and *LPEAT2* overexpressors only a slight increase (up to 20%) in *ATG8a* expression levels was observed in comparison to wild-type plants. Additionally, in *lpeat1* mutant the expression level of *ATG8a* was only slightly changed compared to the control plants—the relative expression was at most 20% lower than in wild-type plants. However, the differences were more pronounced in case of *lpeat2* mutant and *lpeat1 lpeat2* double mutant. In both cases, expression of *ATG8a* was just half as high as in wild-type plants and both differences in expression were significant (Figure 6).

The *ATGa* gene was chosen for analyses as its expression level has been consistently shown to correlate with autophagosomes formation and with an increase in autophagy intensity in Arabidopsis [32]. *ATG8a* gene has also high expression in all the tissues, including leaf tissue investigated by us in this study [30]. Furthermore, we have also shown previously that expression of *ATG8a* gene in the Arabidopsis rosette leaves correlates with activation of autophagy [12]. Moreover, we have previously revealed that the expression pattern of *ATG8a* in Arabidopsis leaves is a good representative of the average gene expression pattern for the whole ATG8 family. This has been shown by qRT-PCR analysis using pairs of primers specific for *ATG8a* and *ATG8s* (i.e., annealing to conserved regions of all *ATG8* genes), respectively [33].

ATG8 protein levels in studied Arabidopsis lines were evaluated by Western blotting using anti-APG8a/ATG8a antibody (abcam). Importantly, this antibody recognizes all nine members of Arabidopsis ATG8 family [30]. The protein extracts (contained 10 µg of total proteins) were separated on NuPAGE 4–12% Bis-Tris-Gel. On these gels, all ATG8 proteins were separated as one distinct band with approximately 100 kDa molecular weight (Figure S2). The same extract (only with another loading buffer and different denaturation temperature and time) separated on urea gels (example on Figure S6) gave several bands of different proteoforms of ATG8, however these proteoforms usually had lower molecular weight than 100 kDa. We cannot explain why on NuPAGE 4–12% Bis-Tris-Gels all of ATG8 proteoforms migrated to one band. However, we found the separation of the protein extracts on such gels very useful for the measurement of total ATG8 protein levels in the plant extracts. The replacement of mentioned above antibody with anti-ATG8 antibody from another company (Agrisera, Sweden), did not result in recognition of this ATG8 proteins aggregate on NuPAGE 4–12% Bis-Tris-Gels (Figure S4B) in spite of the higher amount of protein used for gel separation and the higher antibody concentration used. One must note however, that “Agrisera” uses only a fragment of ATG8 protein as immunogen for preparation of this antibody while “abcam” uses the full length of ATG8 protein for preparation of anti-APG8a/ATG8a antibody (manufacturers’ data). Thus, it is possible that a fragment of ATG8 protein recognized by anti-ATG8 “Agrisera” antibody is not available in such aggregate (band of approximately 100 kDa) for cross-reactivity with “Agrisera” antibody.

**Figure 7:**
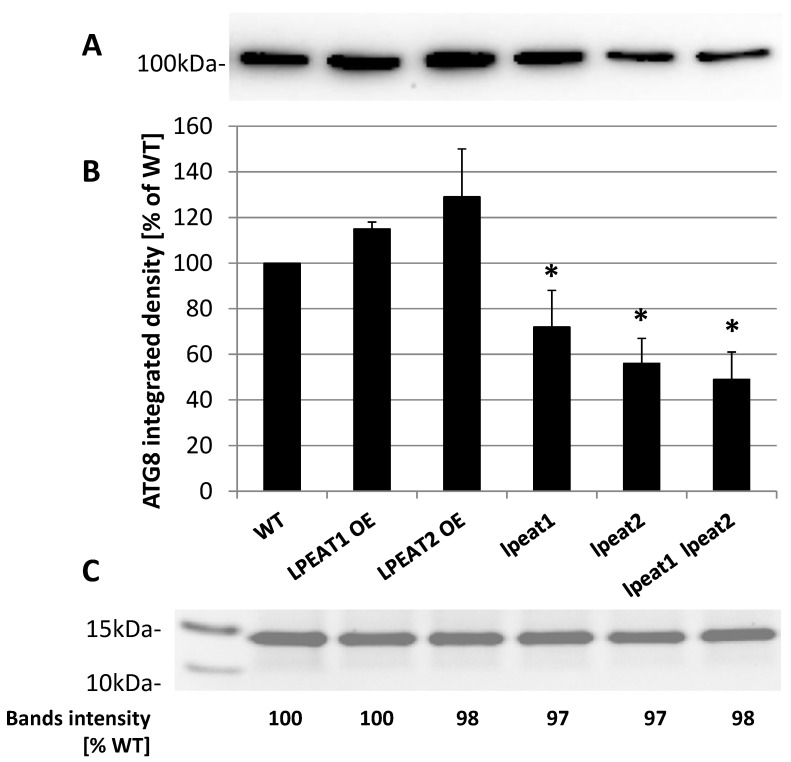
(**A**) AGT8-selected western blot analysis of the total protein extracts from leaves (0 DAF) of wild-type Arabidopsis plants (Col-0) and *LPEAT1* and *LPEAT2* overexpressors, *lpeat1* and *lpeat2* single mutants and *lpeat1 lpeat2* double mutant grown in soil under standard conditions. The blot was incubated with polyclonal antibodies (anti-APG8a/ATG8a (abcam)) against AtATG8 protein. Equal amounts of protein (10 µg) were loaded and separated on NuPAGE 4–12% Bis-Tris-Gel. (**B**) ATG8 band average intensity (means ± SD) from 4 to 5 independent experiments as a percentage of WT. Asterisk indicates significant difference compared with LPEAT1 OE in “mean difference two sided test” at α = 0.05. (**C**) Fragment of selected gel with stained protein bands localized between 15 and 10 kDa and “Image Lab 6.01” analyses of the intensity of these bonds.

**Figure 8:**
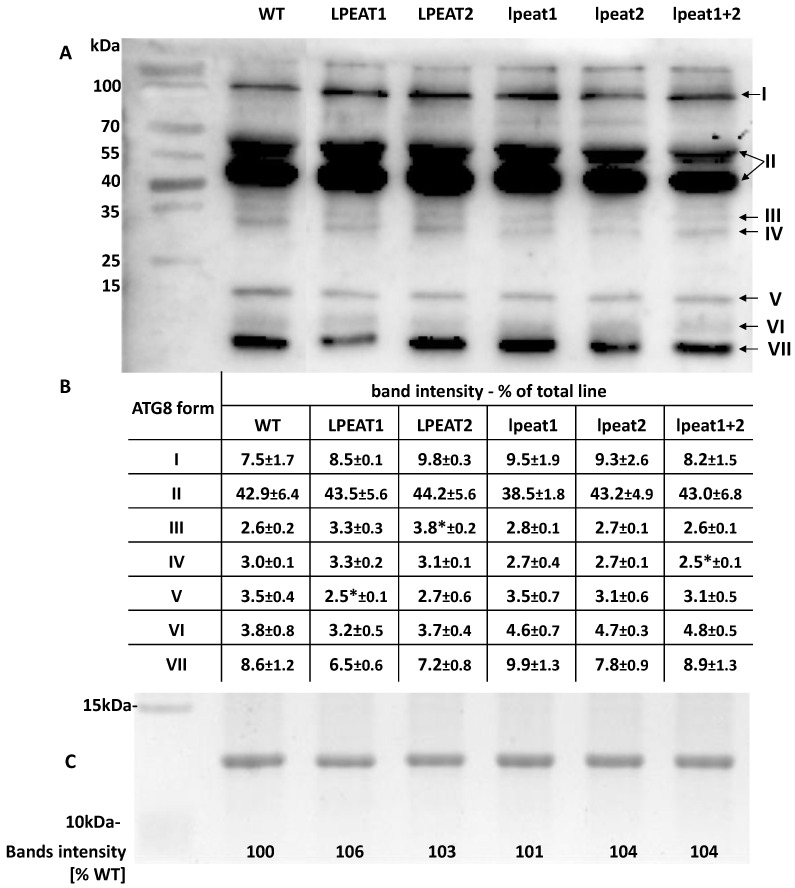
Relative amount of different form of ATG8 proteins in total protein extracts from leaves (0 DAF) of wild-type Arabidopsis plants (Col-0) and *LPEAT1* and *LPEAT2* overexpressors, *lpeat1* and *lpeat2* single mutants and *lpeat1 lpeat2* double mutant grown in soil under standard conditions. (**A**) Selected western blot analysis on urea gel; equal amounts of protein (10 µg) were loaded and separated on the urea gel. The blots were incubated with polyclonal antibodies (anti-APG8a/ATG8a (abcam)) against ATG8 protein. (**B**) Bands intensity determined by “Image Lab 6.01” program. Means ± SD are presented (*n* = 3). Asterisk indicates significant difference compared with WT in “mean difference two sided test” at α = 0.05. (**C**) Fragment of selected gel with stained protein bands localized between 15 and 10 kDa and “Image Lab 6.01” analyses of the intensity of these bonds.

The detected total levels of ATG8 protein among the wild type and transgenic lines corresponded to some extent with *ATG8a* expression levels (Figure 7; confer Figure 6). In extracts of leaves of *LPEAT1* and *LPEAT2* overexpressors the total amount of all ATG8 protein was slightly higher than in wild-type plants (approximately 118 and 125 %, respectively). In *lpeat1* mutant the total amount of ATG8 protein was about 30 % lower than in the wild-type plants, in *lpeat2* mutant about 42 % lower and in *lpeat1 lpeaat2* double mutant about 50% lower (Figure 7). Similarly to NBR1 also in case of ATG8, some gels were stained directly after separation with “commasie brillant blue” (example of full gels on Figures S3, S7 and S9). The bands intensity on these gels (measured with “Image Lab 6.01” program) were very similar in all extracts (examples on Figure 7 and Figure 8).

### 2.4. Relative Amount of Different Forms of ATG8 Proteins in Rosette Leaves of Wild Type Arabidopsis Plants and Plants with Knocked-Out or Overexpressed LPEAT

To investigate whether overexpression or knocking-out of LPEATs encoding genes affects post-translational modification of ATG8 and, in particular, its conjugation with PE, we performed a series of Western blot analyses using urea-containing gels. These gels, contrary to NuPAGE 4–12% Bis-Tris-Gel, revealed several proteoforms recognized by anti-APG8a/ATG8a antibody (abcam). These kinds of blots from plant materials have not been presented previously. Therefore, we have named these proteoforms of ATG8 using Roman numerals starting with the one of highest molecular weight. In order to approximate their real form we have compared their localization on the gels with the data presented by Nakatogawa et al. [34] and Nath et al. [35]. They used yeast and animal analogues of ATG8. As molecular weight of these analogues does not differ much from the plant ATG8, the speed of migration of different plant proteoforms of ATG8 on gels should also be similar. Furthermore, to unambiguously identify ATG8-PE on the Western blots we took advantage of *ATG5* knockout and overexpressor mutants (Figure S6), which show, respectively, a lower and increased accumulation of ATG8-PE [33].

The most abundant form of ATG8 was a form II localized on the gels as two closely adjacent bands with molecular weight of approximately 45 and 65 kDa. Nakatogawa et al. [34] detected in a similar place on the gels also two closely adjacent bands of ATG8xATG3 conjunction. This form accounted for approximately 39–44% of all ATG8 forms (Figure 8). The second most abundant form was proteoform I with molecular weight of around 100 kDa. Nakatogawa et al. [34] identified the ATG8 proteoforms with molecular weight higher than 90 kDa as [ATG8-PE]^n^. The relative amount of proteoform I ranged between 7.5 and 9.8% of all ATG8 forms. The proteoform VII identified by us as ATG8-PE monomer base on molecular weight and our data with *ATG5* overexpressor and *atg5* knockout (Figure S6) constituted (similarly to proteoform I) 6.5 to 9.9% of all ATG8 forms. The proteoform VI localized just above ATG8-PE monomer constituted 3.2 to 4.8% of all ATG8 forms and proteofom V (localized just above proteoform VI) accounted for 2.5 to 3.5%. Base on the literature data [34,35] one of these proteoform is free ATG8. Other distinct identifiable ATG8 forms were proteoform III—2.5 to 3.3% and proteofrom IV—2.6 to 3.8%; localized on the gels as a bands of about 35 and 30 kDa, respectively. The remaining ATG8 forms (measured as signal coming from antibodies “anti-APG8a/ATG8a—abcam” connection with separated proteins) were observed as a smear or very faint bands (Figure 8). Between the tested Arabidopsis lines differences in relative amounts of various forms of ATG8 were neither big nor usually statistically significant. We have to note, however, that *LPEAT* overexpressors contained up to 25% more of total ATG8 proteins and *lpeat* knockouts up to 50% lower amount of total ATG8 proteins compared to the wild-type plants (Figure 7). Thus, although the relative amounts of different forms of ATG8 are similar in all the tested Arabidopsis lines, we have to consider that the absolute amounts of these forms are, like the total amount of ATG8 proteins, higher in overexpressor and lower in the tested knockouts compared to wild type plants. 

The replacement of the above mentioned antibody with anti-ATG8 antibody from another company (Agrisera, Sweden), gave somewhat similar results to those obtained with anti-APG8a/ATG8a (abcam). Similarly to the data presented above, the most abundant proteoform was named on immunoblots with “abcam” antibody as proteoform II. However, the cross-reactivity of this antibody with other proteoforms of ATG8 was generally lower than obtained with the “abcam” antibody. The exception was cross-reactivity with the proteoform, named previously as proteoform VI (Figure S8).

We have to note, however, that both anti-ATG8 antibodies were polyclonal antibodies and some of the detected bonds could be a result of non-specific crosslinking of these antibodies with proteins other than ATG8. However, the observed lack of differences in relative amount of the detected bands (effects of crosslinking of used antibodies with separated proteins) clearly indicates that overexpression or knocking-out of LPEATs encoding genes does not affect post-translational modifications of ATG8. 

### 2.5. Ultrastructure of Cells of Wild Type Plants and Lpeat Mutants of A. thaliana 

**Figure 9:**
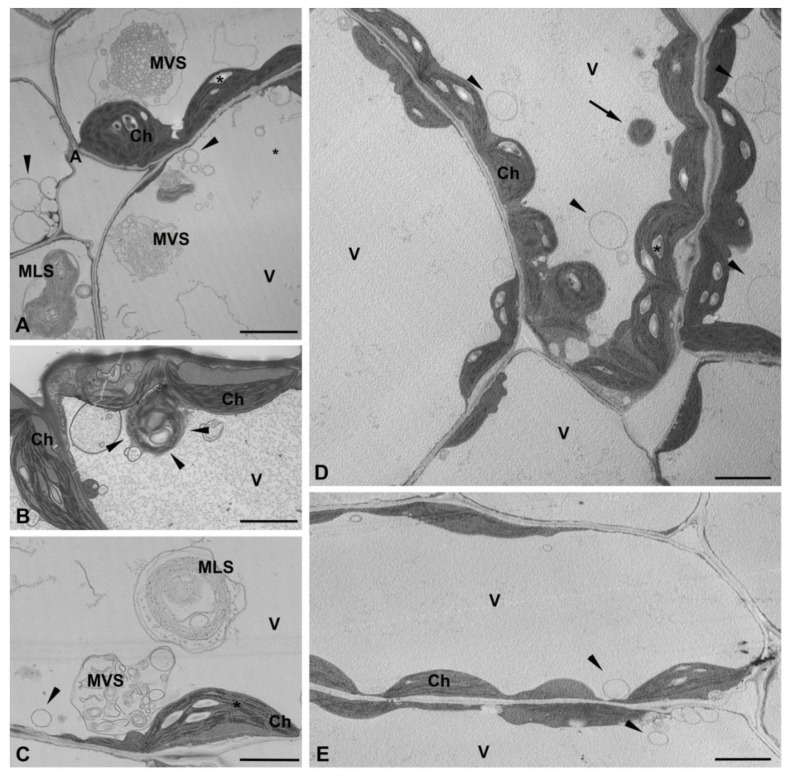
Ultrastructure of mesophyll cells of wild type (**A**–**C**) and *lpeat1 lpeat2* double mutant (**D**,**E**) of *A. thaliana*. (**A**) In vacuole there are numerous membrane structure i.e.,: multivesicular structures (MVS), multilamellar structure (MLS), and single-membrane-bounded vesicles (arrowheads); (**B**) Chloroplast in vacuole (arrowheads) with disturbed thylakoids and concentric membranes (arrowheads); (**C**)The MVS with bigger and less regularly shaped vesicles and MLS with less packed membranes in comparison to similar structures on A; (**D**,**E**) Single-membrane-bounded vesicles (arrowheads) in the vacuoles of the *lpeat1 lpeat2* double mutant; (**D**) In the upper cell a structure reassembling Rubisco-containing bodies (RCBs) (arrow). Ch—chloroplast, V—vacuole, star—starch; Scale bar, 2 μm.

For ultrastructural analysis leaves from 3–4 plants (3 leaves/plant) of wild type (WT) and *lpeat* mutants of *A. thaliana* were used. From the mixture of fragments of central part of leaf blades near the central vascular bundle 10–12 blocks were prepared out of which 5–7 were selected for ultrathin section preparation. In each section up to a dozen palisade mesophyll cells were detected. At least 30–50 cells of palisade mesophyll from each tested Arabidopsis line were analyzed and representative photos from wild type and *lpeat1 lpeat2* double mutant are presented in Figure 9.

In mesophyll cells of leaves of both plant types chloroplasts in thin layer cytoplasm are present along the cell wall and the center of the cell is filled with a large vacuole (Figure 9A–E). In the vacuoles of WT plants numerous membrane structures are present. Among them multivesicular structures (MVS) consisting of a large number of small vesicles surrounded by a single membrane (Figure 9A,C). The size of these vesicles in some of the MVSs was larger and irregular, which may indicate their degradation (Figure 9C). Multilamellar structures (MLS) were the second type of structures present in the vacuoles. They contained several concentric membrane layers, surrounded by a single outer membrane (Figure 9A,C). There were MLSs with more packed layers (Figure 9A) and looser membrane layers (Figure 9C). In the vacuole of WT plants, chloroplasts were also frequently observed. However, they were rounder and the thylakoids system was strongly disturbed. The membranes inside these chloroplasts were arranged concentrically similar to the MLSs (Figure 9B). Additionally in the vacuoles of WT plants a small single-membrane-bounded vesicles were also present (Figure 9A–C) which could have been formed by inward budding of cytoplasm into vacuoles during the microautophagy process. Further analysis of thirty of such mesophyll cells revealed that 34% contained MVSs, 25% MLSs, 55% a degraded chloroplasts, and 67% a small single-membrane-bounded vesicles in their vacuoles (Table S2).

There are significantly fewer cells containing membrane structures in the vacuoles of *lpeat1 lpeat2* double mutant than in the vacuoles of WT plants. The cells do not have at all or have just a single MVS or MLS structure. However, the number of single-membrane-bounded vesicles (a result of microautophagy) is similar to that in the vacuoles of WT plants (Figure 9C,D). Additionally a structure reassembling Rubisco-containing bodies (RCBs) was present. Among 32 analyzed cells, only 12% contained MVSs, 15% MLSs, 42% a degraded chloroplasts, and 64% a small single-membrane-bounded vesicles in their vacuoles (Table S2). The structures of mesophyll cells of leaves of *lpeat2* mutant were similar to those of *lpeat1 lpeat2* double mutant (data not presented).

### 2.6. Lifespan of Wild-Type Arabidopsis Plants and Plants with Knocked-Out or Overexpressed LPEAT

Autophagy is involved in the regulation of plant senescence. As reported previously [12], *atg5* and *atg7* mutants displayed enhanced senescence of fully-expanded leaves. To address phenotypic consequences of LPEAT-mediated modulation of autophagy, we compared phenology of wild type and *lpeat* knockout and *LPEAT* overexpressing plants using previously reported procedure [12].

**Figure 10:**
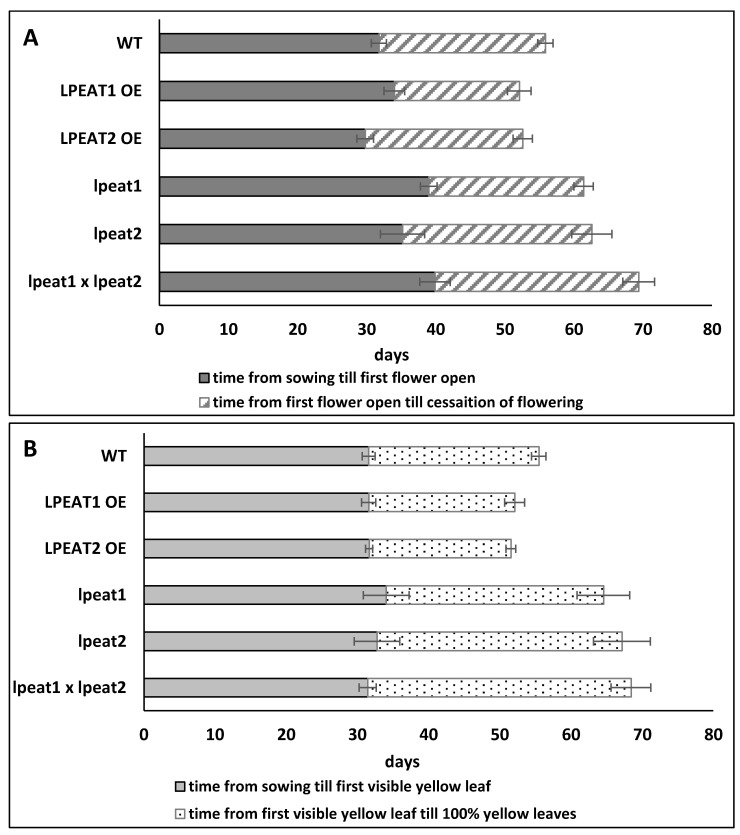
Length of major stages of life cycle of Arabidopsis plant of wild-type, *lpeat1* mutant, *lpeat2* mutant and *lpeat1 lpeat2* double mutant as well as *LPEAT1* and *LPEAT2* overexpressors growth under standard conditions. Panel (**A**) represents the flowering time. Panel (**B**) represents rosette senescence of *A. thaliana.* Presented are average values and standard deviation (*n* ≥ 3).

**Figure 11:**
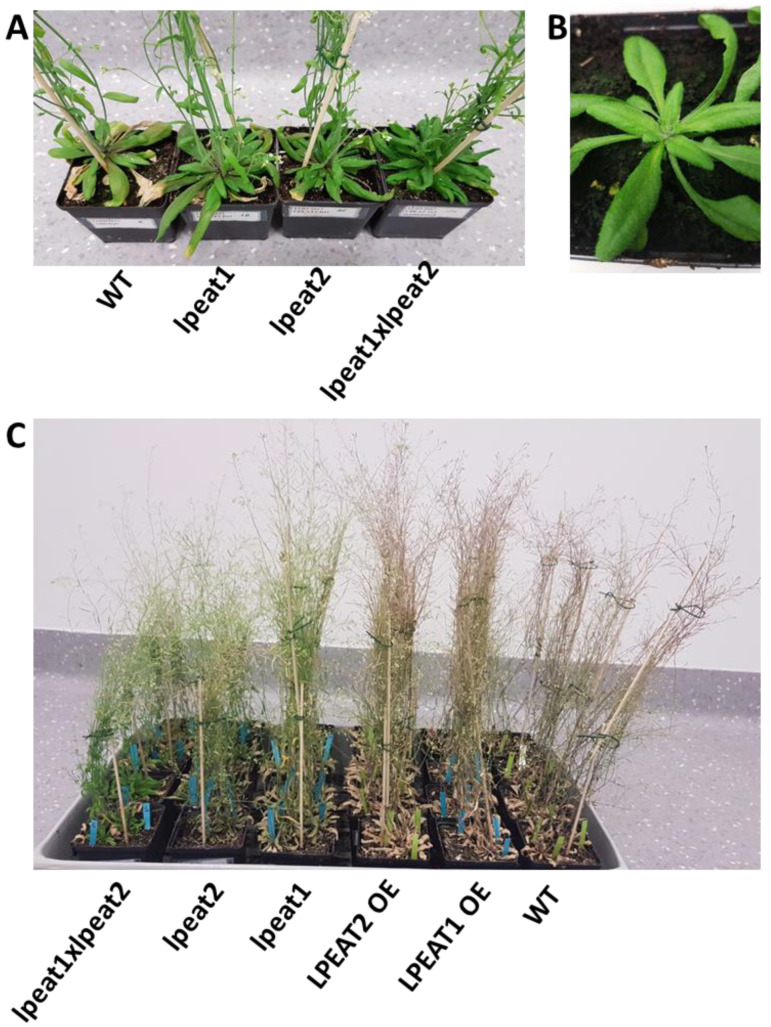
Morphology of wild-type plants of Arabidopsis (Col-0), *lpeat1* mutant, *lpeat2* mutant and *lpeat1 lpeat2* double mutant as well as *LPEAT1* and *LPEAT2* overexpressors grown in soil in standard conditions. The *lpeat1* mutant, *lpeat2* mutant and *lpeat1 lpeat2* double mutant exhibit a delay in senescence in comparison to wild type (panel (**A**,**C**)). Panel (**B**) represents rosette from the wild type plant at 0 days after flowering (0 DAF). Pictures were taken 34 (**A**) and 53 (**C**) days after sowing.

The length of major stages of the life cycle in *LPEAT1* overexpressor and *LPEAT2* overexpressor was decreased compared to WT and *lpeat’s* knockout lines (especially *lpeat2* and *lpeat1 lpeat2* double mutant). The *LPEAT1* and *LPEAT2* overexpressors did not differ from the WT plants in terms of formation of fully expanded cotyledons (data not shown), onset of flowering (first flower open day) or first yellow leaf visible (Figure 10 and Figure 11). However, completion of rosette senescence (i.e., period of time from the first yellow leaf visible until complete rosette yellowing) of *LPEAT1* overexpressor and *LPEAT2* overexpressor, was faster by about 14 and 17%, respectively, compared to WT. Flowering period (time from the first flower open till cessation of flowering) was also shorter by about 25 and 5% in *LPEAT1* overexpressor and *LPEAT2* overexpressor, respectively (Figure 10).

Consistently with our data, the length of major stages of the life cycle in *lpeat* knockouts was longer and the plant development was delayed compared to WT or *LPEAT* overexpressors. In T-DNA knockouts the first flower buds were seen 4–5 days later than in WT and as a consequence, these plants started to bloom 6–8 days later compared to the wild type. The period of time from the first flower opening till cessation of flowering was about 14% longer in *lpeat2* mutant and about 22% longer in *lpeat1 lpeat2* double mutant than in the wild type plants. The rosette leaves also stayed green for a longer time and the senescence process took place slower in *lpeat* mutants compared to WT. Period of time from the first yellow rosette leaf visible (start of senescence) till complete rosette senescence (100% yellow leaves) was increased by about 28% in *lpeat1* mutant, 40% in *lpeat2* mutant and about 46% in *lpeat1 lpeat2* double mutant compared to WT (Figure 10 and Figure 11). In short, all the major life cycle stages, including senescence, were prolonged in the *lpeat* mutants compared to WT. The future studies are needed to confirm if such changes in life cycle stages will be observable also during the cultivation of tested mutants in various stress conditions.

## 3. Discussion

The presented studies describe experiments that were carried out to expand current understanding of the mode of action of LPEATs. Furthermore, the experiments described herein provide basic knowledge concerning subcellular localization of LPEAT proteins.

### 3.1. Subcellular Localisation of LPEAT1 and LPEAT2

Confocal microscopy studies showed that LPEAT1 and LPEAT2 have different subcellular localization. That is, the localization of LPEAT2 to the Golgi and late endosomes is distinct from that of LPEAT1, which was localized exclusively to the ER. It is uncertain whether late endosomes or the Golgi are the *bona fide* residence of LPEAT2 in the cell because of the dynamic flux of membranes between these and other related compartments within the endomembrane system in plants. Further, we cannot rule out the possibility that the localization of LPEAT2 to late endosomes is due, at least in part, to its (transient) overexpression, i.e., the additional ectopically-expressed LPEAT2 protein may have swamped the Golgi and, therefore, LPEAT2 accumulates partially downstream in late endosomes or *vice versa*.

Based at least on that localization data presented in this study we cannot rule out the possibility that LPEAT2 (or LPEAT1) is involved in the formation of the autophagosomal membranes (lipids). The localization of the proteins to compartments in the endosomal pathway certainly puts them in the right place where they could potentially participate in the autophagosome formation. There is also some dispute as to which organelles serve as the template for autophagosomal formation in plant cells, although at least one recent paper points to the ER [36]. The observation that LPEAT2 (and LPEAT1) is not localized to autophagosomes is not entirely unexpected even if they are involved in regulating autophagy. If LPEAT2 (or LPEAT1) are involved in this process in terms of autophagosome formation, one would expect that the protein(s) would be localized to the ‘donor’ organelle membrane and not to the autophagosome itself. The localization of LPEAT1 and 2 to ER and Golgi and endosomes, respectively, is thus not contradicting a possible role in regulating autophagy activity.

### 3.2. Autophagy Markers Levels

The potential involvement of LPEAT in regulation of autophagy intensity in Arabidopsis was evaluated based on the content of two proteins connected with autophagy: NBR1 and ATG8 in leaves of wild-type plants and plants overexpressing *LPEAT1* or *LPEAT2* as well as in *lpeat1* mutant, *lpeat2* mutant, and *lpeat1 lpeat2* double mutant.

NBR1 works as a cargo receptor. At first the toxic protein aggregates are ubiquitinated, and then NBR1 is recruited to the ubiquitinated substrates. This is followed by interaction with ATG8, leading to the formation of autophagosomes around the cargo [37]. The NBR1 protein degrades together with the autophagosomes, thus when authophagy is intensive, the NBR1 level is low and conversely it is elevated when the autophagy intensity is low. Thus, the obtained results suggest that in *lpeat1* mutant, *lpeat2* mutant and *lpeat1 lpeat2* double mutant the intensity of autophagy could be lower than in the wild-type plants.

The amount of NBR1 protein in the tested mutants is difficult to precisely evaluate as we could use only immune-assays for its quantification which give a semi-quantitative data. However, multiple immune-assays suggest that the highest concentration of this protein was in *lpeat1 lpeat2* double mutant followed by *lpeat2* mutant and *lpeat1* mutant. These results correlate well with the intensity of morphological effects caused by the mutations [5]. However, it is open to discussion if the diminished intensity of autophagy is a cause of the impairment of plant growth or whether other factors are responsible for growth restriction and the diminished intensity of autophagy is a side effects with no real influence on the plant development. Our plants were grown in optimal growth conditions and earlier works showed that inhibition of autophagy intensity by knocking out of the genes connected with the autophagy (e.g., *ATG7*, *ATG9,* or *ATG5*) have only a small effect on Arabidopsis grown in optimal conditions. The effects, however, become much more prominent in stress conditions [30,31,38]. The NBR1 level in plants overexpressing *LPEAT1* or *LPEAT2* generally did not differ from that recorded for wild-type plants, indicating that intensity of autophagy was similar in both types of plants. Thus, the more vigorous growth compared to the wild type of overexpressors [5] seems to be not related with the intensity of authophagy.

The ATG8 proteins are acting as starters for autophagosomes formation and can be also involved in membranes intracellular trafficking processes. During various stress conditions, up-regulation of ATG8 encoding genes has been observed in animal and fungi [39]. Enhanced expression of *ATG8a* gene has been also observed in Arabidosis plants grown under low light intensity stress. In this case elevated expression of *ATG8a* gene was well correlated with increased intensity of autophagy (estimated via the NBR1 level measurement) in the leaves [12]. However, stress conditions were also shown to upregulate expression of the *ATG8a* gene in Arabidopsis mutants (e.g., knockout of *ATG5* or *ATG7* genes) with impaired autophagic flux [12,30]. Thus, in this case the environmental conditions regulate the expressions of ATG8 encoding gene. Our experiments, however, seem to indicate that the levels of the LPEAT encoding genes expression (and their products) affect also the expression of the *ATG8a* gene. The inhibition of the expression level of *ATG8a* gene was especially strong in *lpeat1 lpeat2* double mutant followed by *lpeat2* mutant. Some inhibition also occurred in the *lpeat1* mutant. In leaves of plants overexpressing *LPEAT1* or *LPEAT2* the average expression level of *ATG8a* gene was slightly (20 and 15%, respectively) higher than in control plants. Thus, the expression level of *ATG8a* gene strongly correlates with the intensity of morphological effects caused by the mutations [5]. In addition to the measurements of the *ATG8a* gene expression levels we also performed the immune-assays determining the ATG8 protein levels in leaves of the tested mutants. The results have shown that the levels of ATG8 protein in the leaves of tested Arabidopsis lines exhibit a similar pattern to that of *ATG8a* gene expression. One exception was *lpeat1* mutant in which ATG8 protein level was more reduced than expression of *ATG8a* gene. In the assays we used ATG8a antibody, however, this antibody can recognize all nine members of ATG8 protein family thus we see the level of all ATG8 proteins. This means that also genes encoding other ATG8 isoforms could be regulated in a similar way to the gene encoding ATG8a protein with exception of *lpeat1* mutant were other *ATG8* genes seems to be more strongly inhibited. The ATG8 proteins are present in the cells in different forms (see Section 2.4). The relative amounts of these forms of ATG8 in most cases did not differ significantly in the tested Arabidopsis lines. Thus, it seems that the overexpression or knocking-out of *LPEAT* genes do not influence the metabolism of ATG8 proteins after their synthesis. Therefore, we hypothesize that the expression levels of *LPEAT* genes and their products may regulate the expression levels of the genes encoding ATG8 proteins affecting indirectly the amount of these proteins in the cells. The decreased levels of all forms of ATG8 in *lpeat* mutants could consequently lower the intensity of autophagy in these mutants. Thus, the expression levels of *LPEAT* genes might be a new way of regulation of autophagy intensity in plants.

### 3.3. Ultrastructure of Mesophyll Cells

Additionally to the assays determining autophagy markers levels we performed the ultrastructural analyses of mesophyll cells of leaves of wild-type plants and *lpeat1 lpeat2* double mutant. In the vacuoles of WT plants we found many more different membrane structures than in vacuoles of *lpeat1 lpeat2* double mutants. Among them multivesicular structures (MVS) and multilamellar structures (MLS) were in a greater number than in vacuole of *lpeat1 lpeat2* double mutants. Moreover, the percentage of the cells containing those structures were significantly higher in WT plants than in *lpeat1 lpeat2* double mutants. The MLSs could be formed as a consequence of chloroplast degradation via macroautophagy. In the vacuole of analyzed plants, we have observed chloroplasts with rounder structures and concentrically arranged membranes of thylakoids system which could be subsequently converted to MLS. Formation of similar structures to MLSs observed in our studies occurs also during the degradation of photo-damaged chloroplasts following their transport to the central vacuole via autophagy [40,41,42]. The MVSs present in the vacuoles resembles cytoplasmic multivesicular bodies (MVB). So far, there is no hard evidence that cytoplasmic MVB could be transported to the vacuoles via autophagy process. However, a crosstalk between autophagosomes and cytoplasmic multivesicular bodies has recently been described [25,42]. The presence of MLSs and MVSs in much higher abundance in vacuoles of WT plants than in the vacuoles of *lpeat1 lpeat2* mutant together with the results of biochemical studies of NBR1 protein levels (which indicated possibility of decreased autophagy intensity in *lpeat* mutants; see chapter above) additionally suggest that those structures (or at least some of them) could results from the autophagy process.

### 3.4. Life Span of Tested Arabidopsis Lines

In earlier works it has been shown that in stress conditions Arabidopsis mutants with impaired autophagy undergo an earlier senescence; the length of the major stages of life cycle is reduced compared to the control [12,30]. It was even shown that such a lifespan reduction also occurs at normal growth conditions [12]. Thus, we expected that our *lpeat* mutants will also display earlier senescence symptoms than the control plants. However, the results were surprising; instead of earlier senescence the *lpeat* mutants showed a prolonged lifespan. It was pronounced especially in the case of “rosette senescence time” which was up to 55% longer in case of *lpeat1 lpeat2* double mutant. A similar tendency was also observed in other parameters characterizing plant development like “flowering time”, “number of days from sowing to flowering cessation”, or “number of days from sowing to total yellow leaves”. In case of plants overexpressing *LPEAT1* or *LPEAT2* some of the stages of the life cycle were reduced in time or were similar compared to the control. Ergo this data indicates that in case of our mutants the intensity of authophagy is probably not the main (if any) factor determining the lifespan of the plants in the standard growth conditions. Others factors seem to play an important role in this process. One of them could be the elevated levels of LPE and LPC in our *lpeat* mutants [5]. It has already been shown that LPE added exogenously delays leaf and fruit senescence in tomato, cranberry and potato [43,44,45].

### 3.5. Conclusions

The mechanisms behind the regulation of plant growth as a result of changes of *LPEAT* genes expression/LPEAT activity seem to be very complex and consist of regulation of PE content in cells, regulation of the content of lysophospholipids in cells, regulation of the intensity of autophagy and probably many other biochemical processes ([5] and presented data). The knock-out of *LPEAT* genes seems to initiate a cascade of biochemical events, however, the sequence of these events as well as their causes need to be elucidated in further studies.

## 4. Methods

### 4.1. Plant Materials and Growth Conditions

In total, 6 lines of Arabidopsis were used in this study: wild-type *Arabidopsis thaliana* (ecotype Columbia-0 [Col-0]), *lpeat1* (GABI_825D08) and *lpeat2* (SALK_016798c) T-DNA insertion mutant lines (all in Col-0 background; originally obtained from Nottingham Arabidopsis Stock Centre, University of Nottingham, UK), *lpeat1 lpeat2* double mutant, and *LPEAT1-* and *LPEAT2*-overexpressing homozygous lines generated in a previous study [5].

All plant lines were grown simultaneously in a growth chamber at 23 °C, 16 h light (120 µE/m^2^ s)/ 8 h dark and relative humidity 60%. Plant growth was monitored, as previously described [12], beginning at seed germination and continuing through to whole-plant senescence and desiccation.

### 4.2. Subcellular Localisation of LPEAT1 and LPEAT2 in BY-2 Cells

Molecular biology reagents and custom oligonucleotides were purchased from Sigma–Aldrich Corporation (refer to Table S1 for sequence information for all primers used in this study). cDNAs encoding the full-length *AtLPEAT1* and *AtLPEAT2* open reading frames (ORF) were amplified (by PCR) from Arabidopsis leaves cDNA using primers AtLPEAT1f and AtLPEAT1r, and AtLPEAT2f and AtLPEAT2r, respectively (Table S1), and verified by automated sequencing. The corresponding PCR fragments were then cloned into pDONR221 ENTRY vector by GATEWAY^®^ technology using BP clonase reaction [46]. The appropriate DNA fragments were subsequently cloned (via the LR reaction) into the pMDC43/mGFP DESTINATION vector [47] containing the ORF of mGFP, followed by a multiple cloning site, and the 35S cauliflower mosaic virus promoter. Other plant expression vectors used in this study have been described elsewhere and included pRTL2/RFP-ATG8a encoding the Arabidopsis ATG8a fused at its N terminus to the RFP [48]; pRTL2/Myc-Sft11 encoding the Golgi-localized Arabidopsis SNARE Sft11 linked at its N terminus to the Myc epitope sequence (consisting of amino acids -EQKLISEEDL- [49]) [50]; and pRTL2/RFP-ARA7 encoding the late endosome-localized Arabidopsis Rab5-related GTPase ARA7 fused to the C terminus of the RFP [51].

*Nicotiana tabacum* BY-2 suspension-cultured cells were maintained and prepared for (co)transformation via biolistic particle bombardment using a Bio-Rad Particle Delivery System 1000/HE, as described previously [52]. Transient (co)transformations were performed using 1–2 µg of plasmid DNA. Following bombardment, cells were incubated for 4–6 h to allow for expression and sorting of the introduced gene product(s) and based on the results of preliminary experiments aimed at ensuring that any potential negative effects due to protein overexpression were diminished, consistent with other studies from our lab involving BY-2 cells serving as model systems for characterizing intracellular protein localization (e.g., [17,53,54,55,56]). Induction of autophagy in BY-2 cells with BTH was carried out in a similar manner to that reported previously for BY-2 cells [57], as well as Arabidopsis cells [58], and according to guidelines for inducing and monitoring autophagy described elsewhere [27,59,60]. Briefly, cells were incubated during and post-bombardment periods in 10 µM BTH (Sigma–Aldrich Canada, Oakville, ON, Canada) or in water only, serving as a mock control.

BY-2 cells were processed for CLSM imaging, including immunostaining and ER staining with ConA conjugated to Alexa 594 (Molecular Probes, Eugene, OR, USA), as previously described [51]. Mouse anti-Myc immunoglobulin G (IgG) in hybridoma medium were obtained from Princeton University Monoclonal Antibody Facility and goat anti-mouse rhodamine red-X IgG were from Molecular Probes. All imaging was carried out using a Leica DM RBE microscope equipped with a 63× Plan Apochromat oil-immersion objective and TCS SP2 scanning head (Leica Microsystems Canada Inc, Richmond Hill, ON, Canada). Excitations and emission signals for fluorescent- or epitope-tagged proteins and ConA fluorescence were collected sequentially as single optical sections in all (co)transformation and ConA-staining experiments, as those described in Gidda et al. [61], and single-labelling experiments showed no detectable crossover at the settings used for data collection. Micrographs shown in figures are representative of the results obtained from analyzing ≥20 independently transformed cells and cell areas from at least two separate experiments (i.e., (co)bombardments).

### 4.3. Determination of NBR1 and ATG8 Protein Levels in Leaves of A. thaliana

NBR1 and ATG8 protein levels were determined by Western blotting using specific antibodies. To extract soluble proteins, 100 mg of rosette leaves (at 0 day after flowering (0 DAF)) were frozen in liquid nitrogen and homogenized in 100 µL of extraction buffer (4 M urea, 10 mM DTT, 1% (*v*/*v*) Triton X-100). After homogenization samples were kept on ice for 30 min followed by centrifugation at 13,000 rpm (Eppendorf “Centrifuge 5415C”) for 5 min. Protein concentration in the supernatants was determined using BCA Protein Assay Reagent (Pierce Chemical, Rockford, IL, USA) according to the manufacturer’s instructions. Aliquots containing 30 or 10 µg protein for immunoblotting of NBR1 or ATG8, respectively, were denatured in loading buffer (4x LDS Invitrogen, Carlsbad, California) for 20 min at 65 °C. Proteins were loaded onto NuPAGE 4–12% Bis-Tris-Gel (Invitrogen), separated using electrophoresis apparatus Xcell II Blot module (Invitrogen) at 200 V and blotted to a nitrocellulose membrane (Invitrogen, LC2001). The membranes were washed with Tris-buffered saline (TBS; pH 7.5) and blocked with 5% milk in TBS overnight at +6 °C.

For immunodetection, membranes were washed twice in TBS for 10 min and then incubated with anti-NBR1 (Agrisera, Sweden) or anti-APG8A/ATG8A (abcam) at a dilution of 1:5000 for 1.5 h at room temperature with agitation. The antibody solution was decanted and the membranes were washed twice for 10 min in TBST (pH 7.5) and twice for 10 min in TBS buffer at room temperature with agitation, followed by incubation with secondary antibodies (goat anti-rabbit IgG horse peroxidase conjugated, Agrisera AB, Vännäs, Sweden) diluted 1:10,000 with 5% milk in TBS for 1 h at room temperature with agitation. The membranes were washed four times for 10 min in TBST and developed with chemiluminescent mixture (BM Chemiluminescence Western Blotting Kit, ROCHE, Basil, Switzerland) according to manufacturer’s instructions, before imaging using a ChemiDoc (BioRad, Hercules, CA, USA) and Quantity One software (Bio-Rad).

To determine the relative amounts of different forms of ATG8, the aliquots of extracts containing 10 µg protein (see above) were denatured in the presence of Laemmli buffer for 10 min at 100 °C, stacked on 4% polyacrylamide gel (containing 0.125 Tris-HCl pH 6.8 and 0.1% SDS) and then separated on 15% polyacrylamide gel (containing 6 M urea, 0.375 Tris-HCl pH 8.8 and 0.1% SDS) using Mini-PROTEAN Tetra Cell system (BioRad). The proteins were blotted to a nitrocellulose membrane (Invitrogen, LC2001), which was blocked overnight at 6 °C with 5% milk in phosphate-buffered saline (PBST), washed twice in PBST for 5 min and then incubated with anti-APG8/ATG8a (abcam) diluted 1:1000 with 1% BSA in PBST for 2 h at room temperature with agitation. Thereafter, the membrane was washed twice in PBST for 10 min and incubated with goat anti-rabbit IgG horse peroxidase conjugate (Agrisera) diluted 1:5000 with 1% BSA in PBST for 1 h at room temperature with agitation. Finally, the membrane was washed three times for 10 min in PBST and the blot developing procedure was conducted as described above.

For immunodetection of ATG8 proteins in tested extracts additionally antibodies “anti-ATG8” from the “Agrisera, Sweden” were used. The immunodetection procedure was as described above for anti-APG8A/ATG8A (abcam), however, higher amounts of proteins were used for gel separation (indicated in figure captions; Figures S4, S5, S8 and S9), and the concentration of antibodies was 2-fold higher.

### 4.4. Measuring ATG8a and NBR1 Expression Levels in Leaves of A. thaliana by q-RT-PCR

Total RNA was extracted from 100 mg of liquid-nitrogen flash-frozen Arabidopsis leaves at 0 DAF using Universal RNA Purification Kit (EURx) and treated with dsDNase (Thermo Fisher Scientific, Waltham, MA, USA) to remove any potential DNA contaminants. cDNA was prepared using Maxima First Strand cDNA Synthesis Kit for RT-qPCR (Thermo Fisher Scientific). Primers for *ATG8a* (*At4g16520*) and *NBR1 (At4g24690*), as well as for reference genes (*EF1ALPHA*-*At5g60390* and *ACT2*-*At3g18780*) expression measurements are included in Table S1. After optimizing and confirming linearity of primer amplification, quantitative PCR experiments were conducted in accordance with the protocol described for PowerUp SYBR Green Master Mix (Thermo Fisher Scientific) in LightCycler 480 (Roche). Values were normalized to *EF1ALPHA* and *ACT2*.

### 4.5. Ultrastructural Analysis of Cells of Leaves of Wild Type Plants and Lpeat Mutants of A. thaliana

From the leaves of four and a half weeks old plants small fragments of central part of leaf blades near the central vascular bundle were cut out and fixed with 4% (*v*/*v*) formaldehyde and 2% (*v*/*v*) glutaraldehyde in phosphate buffered saline (PBS, pH 7.2) for 1 h in an incubator connected to vacuum pump followed by an overnight fixation at 4 °C. After washing with distilled water the leaves were post-fixated in 1% OsO_4_ by 1h. Samples were dehydrated in graduated ethanol concentrations, embedded in Spurr resin (Sigma) according to the standard protocol, and then ultrathin sections were collected on nickel grids. Finally, the sections were stained with 2.5% uranyl acetate (30 min) and 2.5% lead citrate (10 min.) and examined using transmission electron microscopy (TEM; Joel 1010) at 80 kV (modified method according to: Niedojadło and Górska-Brylass [62]).

## Data Availability

The data presented in this study are available on request from the corresponding author. The data are not publicly available due to privacy.

## Supplementary Materials

The following are available online at https://www.mdpi.com/1422-0067/22/6/3006/s1.
