# Supplementary material for: Subcellular Localization of Acyl-CoA: Lysophosphatidylethanolamine Acyltransferases (LPEATs) and the Effects of Knocking-Out and Overexpression of Their Genes on Autophagy Markers Level and Life Span of A. thaliana
Source: Int J Mol Sci. 2021 Mar 16;22(6):3006. doi: 10.3390/ijms22063006 (PMC8000221; doi:10.3390/ijms22063006)
Supplement: Supplementary file 1 [file ijms-22-03006-s001.pdf]

Int. J. Mol. Sci.

Subcellular localization of acyl-CoA:lysophosphatidylethanolamine acyltransferases (LPEATs) and the effects of knocking-out and overexpression of their genes on autophagy markers level and life span of *A. thaliana*

Katarzyna Jasieniecka-Gazarkiewicz[1*], Kamil Demski[1], Satinder K. Gidda[2], Sylwia Klińska[1], Janusz Niedojadło[3], Ida Lager[4], Anders S. Carlsson[4], Elena A. Minina[5], Robert T. Mullen[2], Peter V. Bozhkov[5], Sten Stymne[4] and Antoni Banaś[1]

Supplementary materials

Table S1. List of primers used in the study

Primer name	5' primer sequence 3'
AtLPEAT1f	GGGGACAAGTTTGTACAAAAAAGCAGGCTTCGAATCAGAGCTCAAAGA
AtLPEAT1r	GGGGACCACTTTGTACAAGAAAGCTGGGTCTCATTCTTCTTTCTGATGGAAATC
AtLPEAT2f	GGGGACAAGTTTGTACAAAAAAGCAGGCTTCGCGGATCCTGATCTGTCTTCT
AtLPEAT2r	GGGGACCACTTTGTACAAGAAAGCTGGGTCTTATGTTGGGGCCAAGTCA
EF1ALPHAf qPCR	TGCTCTATGGAAGTTCGAGACC
EF1ALPHAr qPCR	GTGGCATCCATCTTGTTACAACA
ACT2f qPCR	TGGAATCCACGAGACAACCTA
ACT2r qPCR	TTCTGTGAACGATTCCTGGAC
Atg8f qPCR	ATGATCTTTGCTTGCTTGAAATTC
Atg8r qPCR	AGCCTTCTCCACAATCACG
NBR1f qPCR	TGATGAGGATGGGGATGTG
NBR1r qPCR	AGCAGCAGAGTTAGTGGAC

Table S2. Percentage of mesophyll cells containing different membrane structures in their vacuoles.

Analyzed Arabidopsis line	% of cells containing in vacuoles the following membrane structures:			
	MVS	MLS	Degraded chloroplasts	Small single-membrane-bounded vesicles
WT	34	25	55	67
lpeat1 lpeat2 double mutant	12	15	42	64

MVS – multivesicular structures; MLS - multilamellar structures; for analyses 30 cells of WT and 32 cells of *lpeat1 lpeat2* double mutant were used

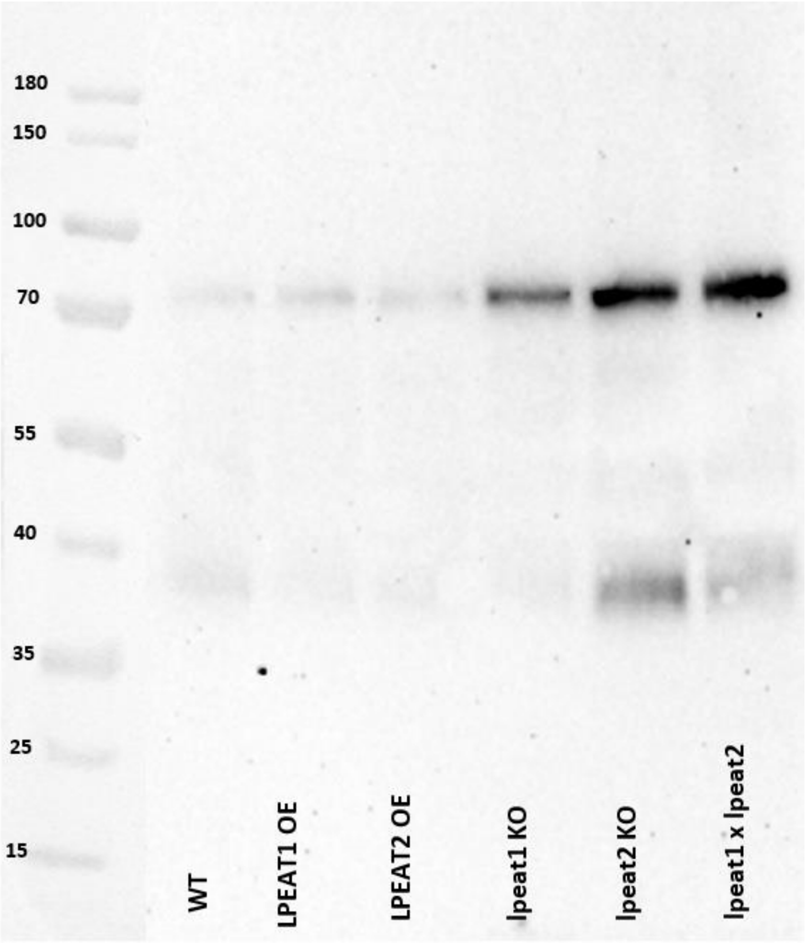

Fig. S1. Selected western blot analysis (complete membrane) of the total protein extracts from leaves (0 DAF) of wild-type plants of Arabidopsis (Col-0) – (WT) and plants of *lpeat1* mutant, *lpeat2* mutant and *lpeat1 lpeat2* double mutant as well as *LPEAT1* and *LPEAT2* overexpressors grown in soil under standard conditions. The blot was incubated with polyclonal antibodies against AtNBR1 protein. Equal amounts of protein (30 μg) were loaded and separated on the NuPAGE 4-12% Bis-Tris-Gel. The ladder with pre-stained protein standards came from the same membrane which was used for incubation with antibodies. Photo of the ladder was combined with the blot.

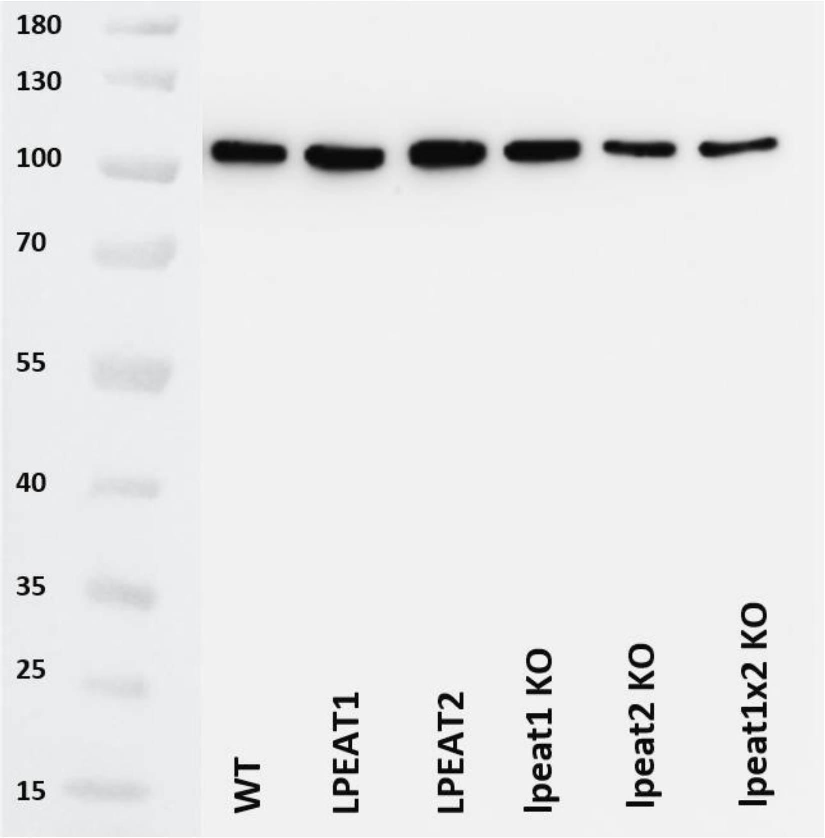

Fig. S2. Selected immunoblot analysis (complete membrane) of the total protein extracted from leaves (0 DAF) of wild type plants of Arabidopsis (Col-0) (WT), *lpeat1* mutant, *lpeat2* mutant and *lpeat1 lpeat2* double mutant as well as *LPEAT1* and *LPEAT2* overexpressors. The blot was incubated with polyclonal antibodies (abcam) against ATG8a protein. Equal amounts of protein (10 μg) were loaded and separated on the NuPAGE 4-12% Bis-Tris-Gel. The ladder with pre-stained protein standards came from the same membrane which was used for incubation with antibodies. Photo of the ladder was combined with the blot.

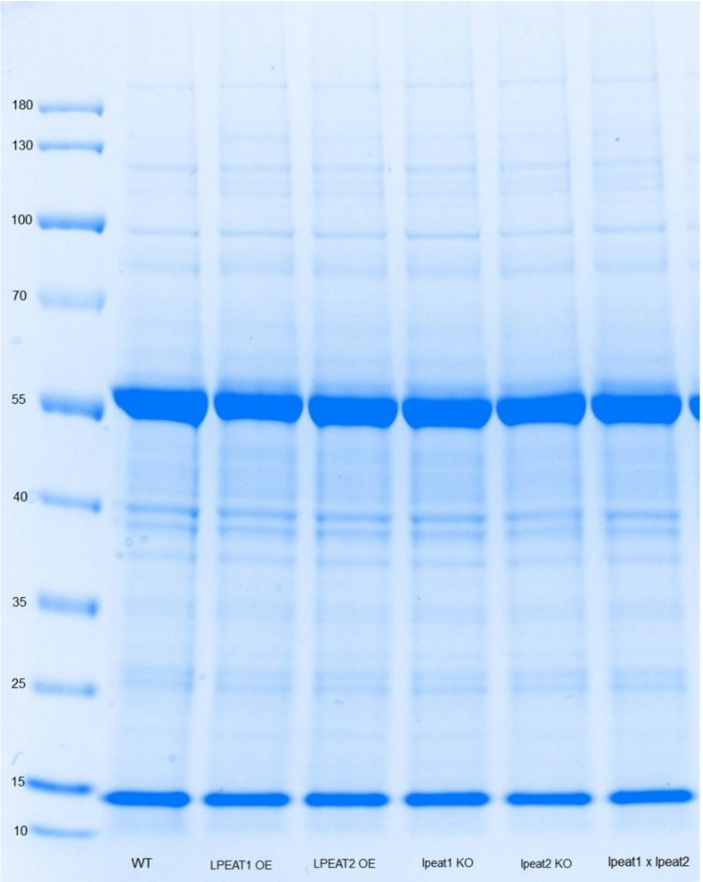

Fig. S3. Total protein extracted from leaves (0 DAF) of wild type plants of Arabidopsis (Col-0) - (WT), *lpeat1* mutant, *lpeat2* mutant and *lpeat1 lpeat2* double mutant as well as *LPEAT1* and *LPEAT2* overexpressors separated on the NuPAGE 4-12% Bis-Tris-Gel. On the gel equal amounts of protein (10 µg) were loaded. The proteins were stained with “commasie brillant blue”.

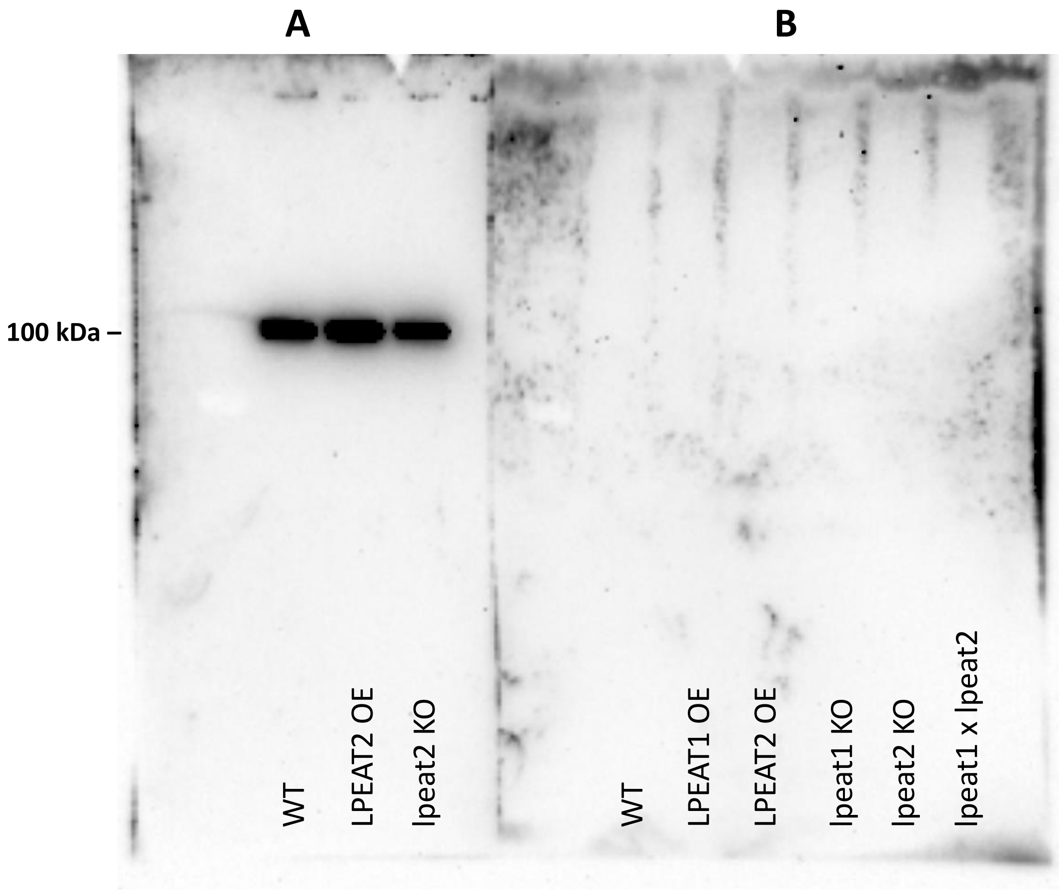

Fig. S4. Immunoblot analysis of the total protein extracted from leaves (0 DAF) of wild type plants of Arabidopsis (Col-0) (WT), *lpeat1* mutant, *lpeat2* mutant and *lpeat1 lpeat2* double mutant as well as *LPEAT1* and *LPEAT2* overexpressors. The blot was cut for two parts and incubated with polyclonal antibodies against ATG8a protein; "part A" with antibodies from "abcam" and part B" with antibodies from "Agrisera". On the gel: "part A" 10 µg of protein/wells and in "part B" 40 µg of protein/wells were loaded and separated. NuPAGE 4-12% Bis-Tris-Gel was used.

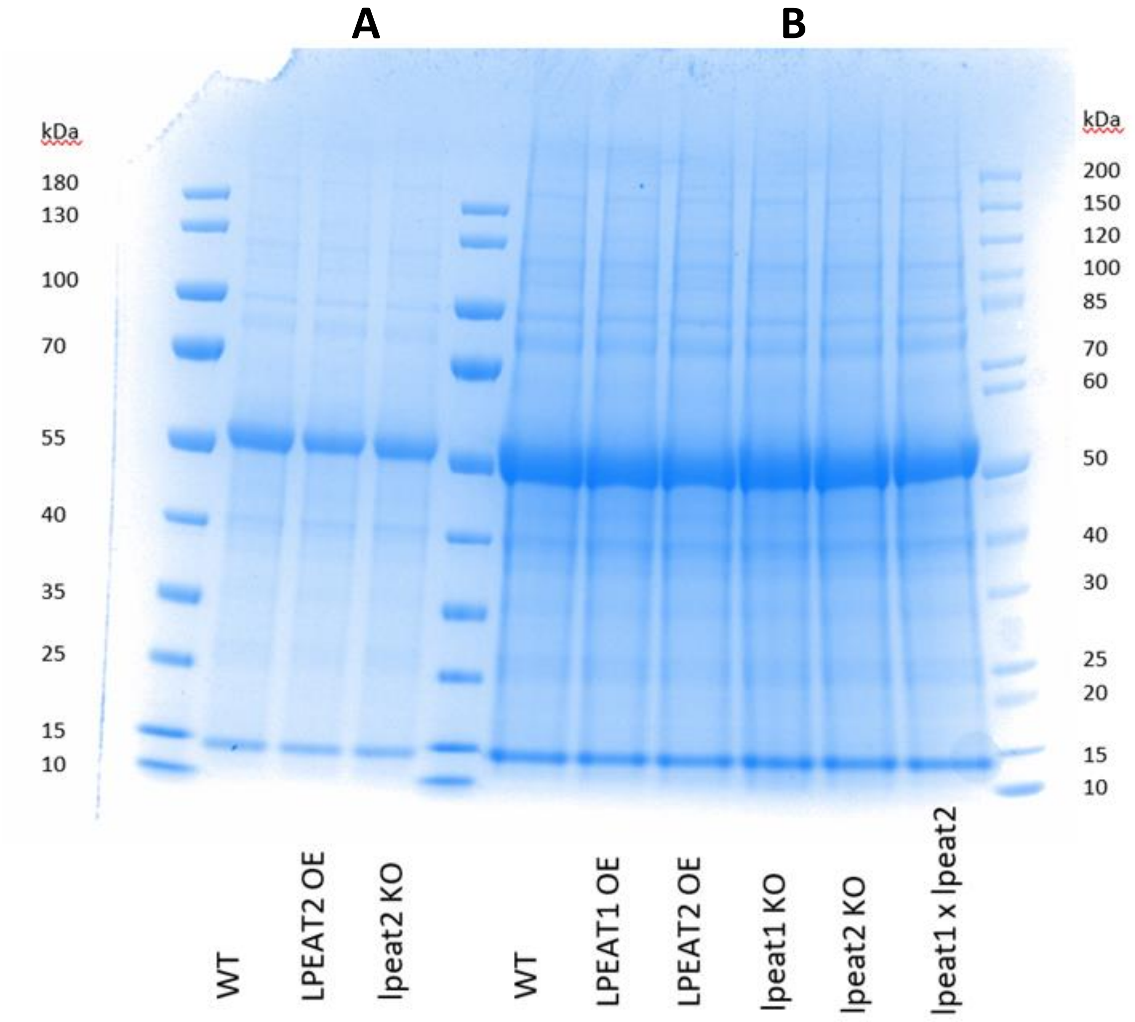

Fig. S5. Total protein extracted from leaves (0 DAF) of wild type plants of Arabidopsis (Col-0) - (WT), *lpeat1* mutant, *lpeat2* mutant and *lpeat1 lpeat2* double mutant as well as *LPEAT1* and *LPEAT2* overexpressors separated on the NuPAGE 4-12% Bis-Tris-Gel. On the gel: "part A" 10 µg of protein/wells and in "part B" 40 µg of protein/wells were loaded. The proteins were stained with "commasie brillant blue".

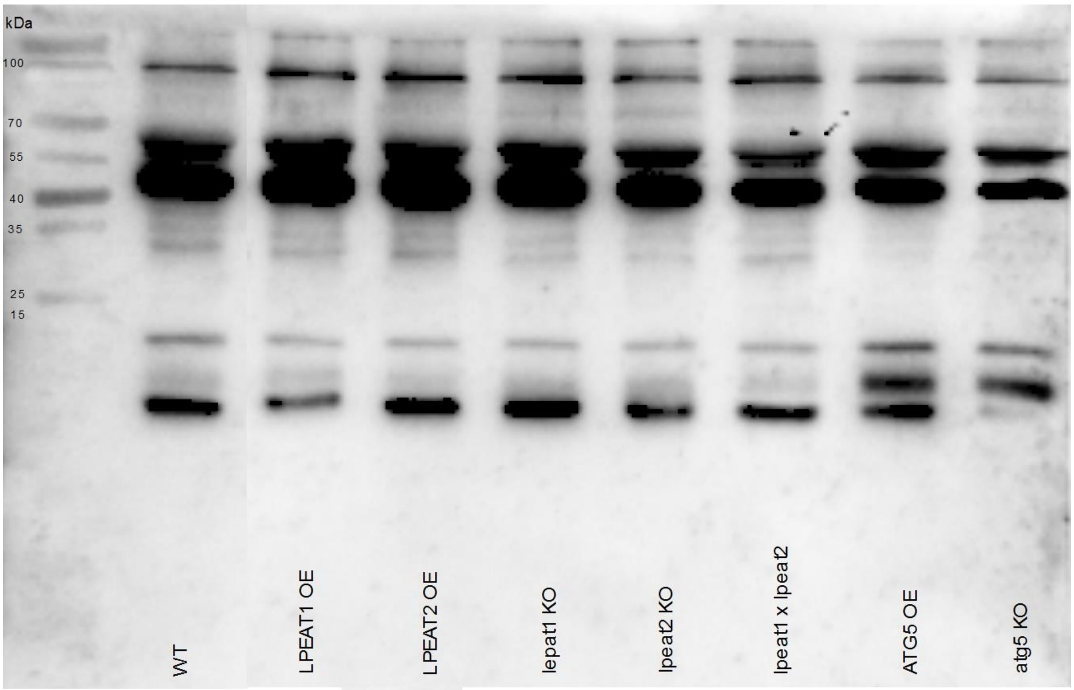

Fig. S6. Selected immunoblot analysis (complete membrane) of the total protein extracted from leaves (0 DAF) of wild type plants of Arabidopsis (Col-0) - (WT), *lpeat1* mutant, *lpeat2* mutant and *lpeat1 lpeat2* double mutant as well as *LPEAT1* and *LPEAT2* overexpressors. Additionally protein extracts form leaves of Arabidopsis *ATG5* OE and *atg5* mutant (0 DAF) were separated. The blot was incubated with polyclonal antibodies against ATG8a protein from "abcam". Equal amounts of protein (10 µg) were loaded and separated on the urea gel. All wells came from the same blot; one well between WT and *LPEAT1* overexpressors was cut off.

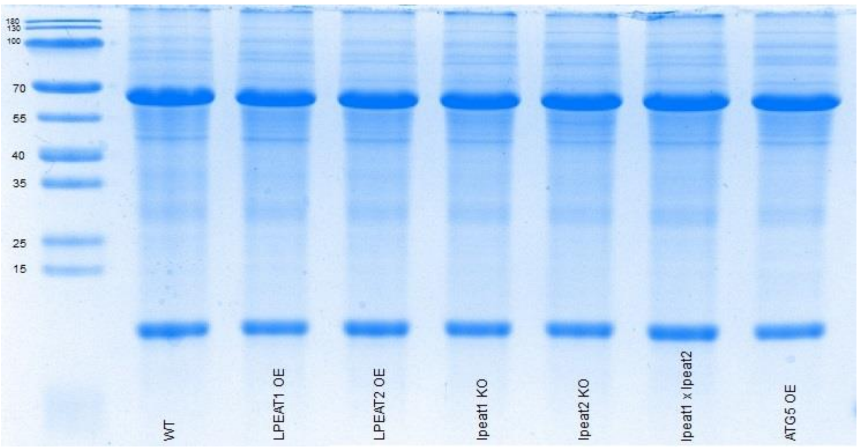

Fig. S7. Total protein extracted from leaves (0 DAF) of wild type plants of Arabidopsis (Col-0) - (WT), *lpeat1* mutant, *lpeat2* mutant and *lpeat1 lpeat2* double mutant as well as *LPEAT1* and *LPEAT2* overexpressors separated on the urea gel. Additionally protein extracts of Arabidopsis *ATG5* OE (0 DAF) were separated. On the gel equal amounts of protein (10 μg) were loaded. The proteins were stained with “commasie brillant blue”.

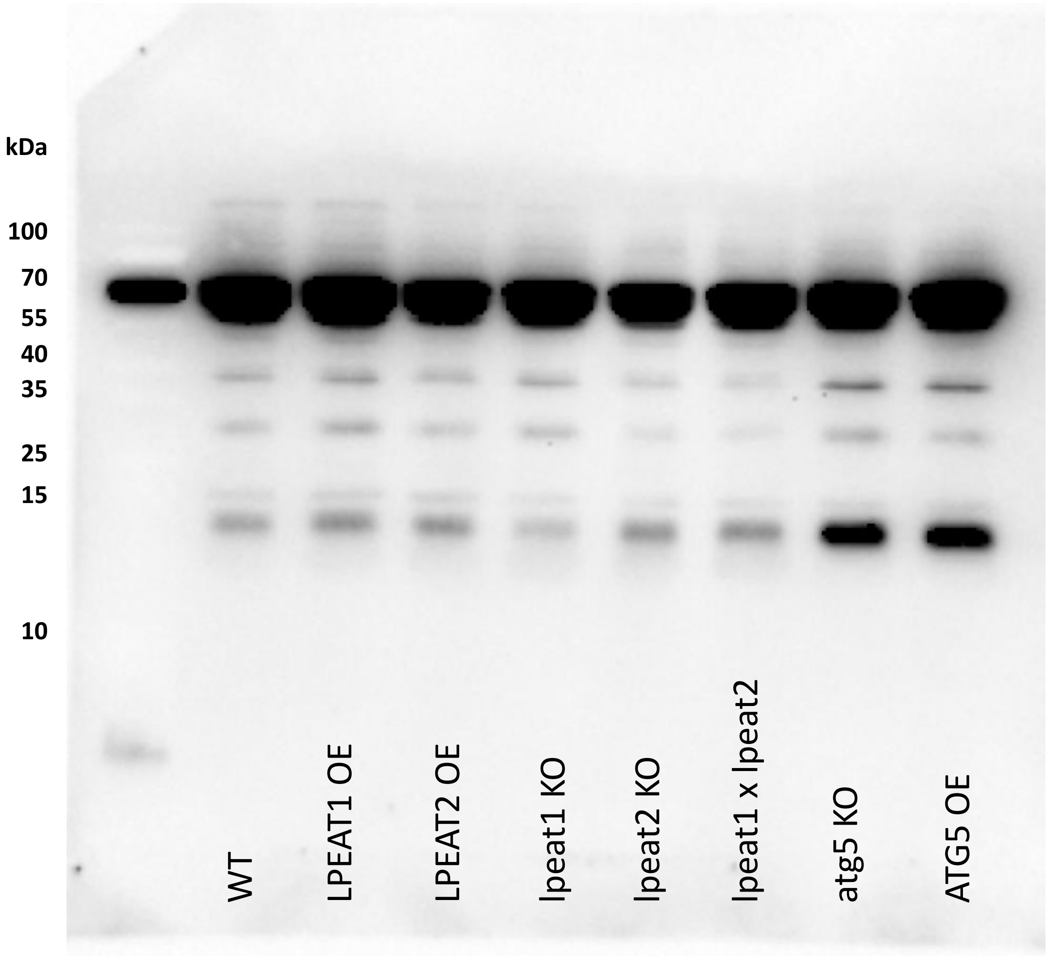

Fig. S8. Immunoblot analysis (complete membrane) of the total protein extracted from leaves (0 DAF) of wild type plants of Arabidopsis (Col-0) - (WT), *lpeat1* mutant, *lpeat2* mutant and *lpeat1 lpeat2* double mutant as well as *LPEAT1* and *LPEAT2* overexpressors. Additionally protein extracts form leaves of Arabidopsis *ATG5* OE and *atg5* mutant (0 DAF) were separated. The blot was incubated with polyclonal antibodies against ATG8a protein from “Agrisera”. Equal amounts of protein (15 µg) were loaded and separated on the urea gel.

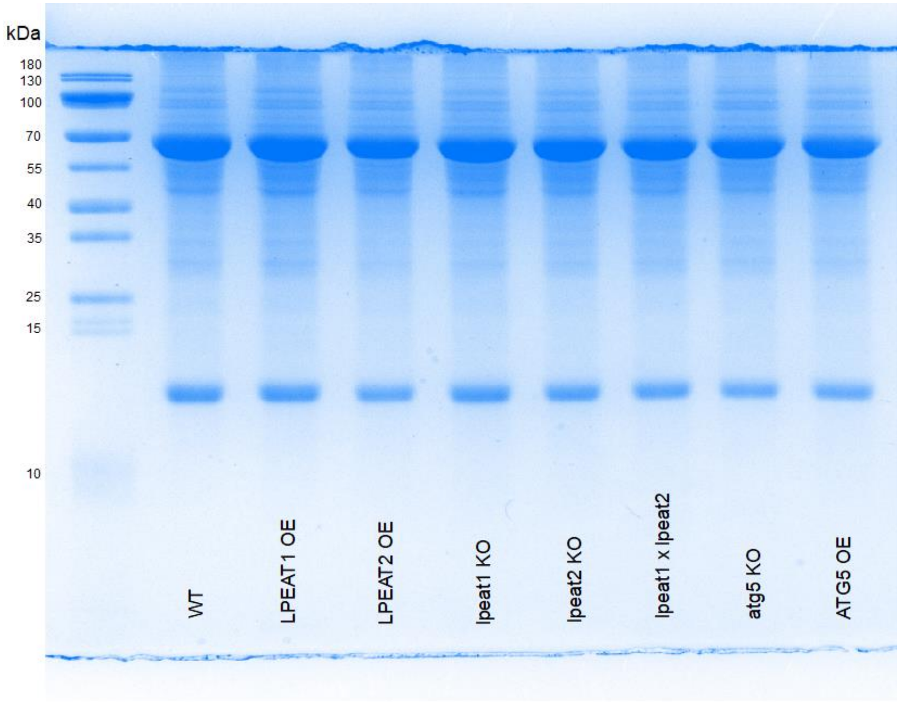

Fig. S9. Total protein extracted from leaves (0 DAF) of wild type plants of Arabidopsis (Col-0) - (WT), *lpeat1* mutant, *lpeat2* mutant and *lpeat1 lpeat2* double mutant as well as *LPEAT1* and *LPEAT2* overexpressors separated on the urea gel. Additionally protein extracts from leaves of Arabidopsis *ATG5* OE and *atg5* mutant (0 DAF) were separated. On the gel equal amounts of protein (15 µg) were loaded. The proteins were stained with "commasie brillant blue".
